# The impact of executive compensation incentive on corporate innovation capability: Evidence from agro-based companies in China

**DOI:** 10.1371/journal.pone.0291517

**Published:** 2023-09-12

**Authors:** Yue Shang, Jian Xu, Jing Li

**Affiliations:** 1 Financial Department, Linyi University, Linyi, China; 2 School of Economics and Management, Qingdao Agricultural University, Qingdao, China; 3 School of Ocean Technology Sciences, Qilu University of Technology, Jinan, China; Universite de Kairouan, TUNISIA

## Abstract

This paper aims to examine the impact of executive compensation incentive on corporate innovation capability by dividing executive compensation incentive into short-term monetary incentive and long-term equity incentive. We also investigate the interaction between the two types of executive compensation incentive. Data are collected from China’s agro-based companies during 2012–2019, and multiple regression analysis is utilized. The empirical results show that short-term monetary incentive has no impact on innovation capability, while long-term equity incentive stimulates innovation capability. Regarding company ownership, the impact of long-term equity incentive in state-owned enterprises is greater than that in private-owned enterprises. In addition, the complementary effect between short-term and long-term compensation incentive has a positive impact on innovation capability regardless of company ownership. The findings of this paper could help agribusiness managers to design the reasonable incentive system to incentivize corporate executives and enhance the capability of independent innovation.

## 1. Introduction

Under the modern enterprise system, the separation of ownership and management results in the principal-agent conflict problem, and how to solve this problem has become a hot issue for scholars at home and abroad [1]. According to Jensen and Meckling [2], supervision and incentive can avoid the unreasonable behaviors of managers, which in turn reduces the principal-agent problem and maximizes shareholder wealth and firm value. Executives hold final responsibility for budgets that affect the firm’s investment in research and development (R&D) [3]. They can allocate more resources to innovation by establishing an innovation-oriented strategy [4]. However, technological innovation has the characteristics of high risk, high investment, and long payback period, which hinders executives’ enthusiasm towards innovation [5]. In order to balance the interests of owners and managers and stimulate management team’s creativity, it is essential to give effective compensation incentives to managers. Short-term monetary incentive and long-term equity incentive are two common incentive methods in modern corporate governance [6]. Short-term monetary incentive includes cash salary and cash bonus, and long-term equity incentive enables executives to hold corporate stocks and links executive compensation with stock prices, which can effectively coordinate the conflict of interest between executives and shareholders [7, 8]. However, the form of compensation incentive could be more important than incentive level in terms of motivating executives to work hard for the shareholders [9]. Therefore, it is necessary to analyze the impact of different forms of executive compensation incentive on corporate innovation capability.

Since the reform and opening-up, China’s agricultural economy has made gratifying achievements. Grain output per unit area has been greatly increased, rural labor force has been gradually transferred, and agriculture has provided a large amount of funds to support industrial development [10, 11]. These achievements cannot be separated from innovation capability of agro-based companies [12]. Although the innovation capability of China’s agro-based companies witnesses an annual improvement, it is still below the national average level. The total amount of innovation inputs and outputs increases slightly, but the growth rate is relatively small [13]. In addition, professional talents in this sector are insufficient, which hinders the sustainable development of modern agriculture [12, 14]. Therefore, it is important for agro-based companies to improve innovation capability and enhance core competitiveness in today’s dynamic business environment.

Among many factors affecting innovation capability of agro-based companies, this paper focuses on the impact of executive compensation incentive. Some scholars believed that compensation incentive is a short-term behavior that generates far less income than long-term investment. When companies pursue more long-term interests, such short-term incentive cannot well promote corporate innovation [15], while equity incentive can play a vital role [16]. Therefore, a reasonable executive compensation structure can achieve the optimal design of incentive compensation and promote innovation capability of China’s agro-based companies.

Domestic and foreign scholars have not reached a consistent conclusion on the relationship between executive compensation incentive and corporate innovation capability. This paper takes agro-based companies listed on the Shanghai and Shenzhen stock exchanges from 2012 to 2019 as the sample to verify the relationship between different forms of executive compensation incentive and corporate innovation capability and compare the differences in the role of short-term monetary incentive and long-term equity incentive. In addition, we also analyze the relationship between them in different types of companies, namely, state-owned enterprises (SOEs) and private-owned enterprises (POEs). Our results reveal that only long-term equity incentive has a positive impact on innovation capability, and its impact in SOEs is greater than that in POEs. In addition, the complementary effect between short-term and long-term compensation incentive has a positive impact on innovation capability regardless of company ownership. By doing so, this paper attempts to contribute to the existing literature by finding out the underlying influence of executive compensation incentive on innovation capability of agro-based companies.

The contributions of this paper are as follows. First, previous studies have mainly focused on the impact of executive compensation incentive on corporate innovation capability in high-tech industries [17], and little has been done in agricultural sector. Unlike other sectors, agricultural sector has its own specificities due to high dependency on natural factors [10]. This paper attempts to fill this gap by using data from agro-based listed companies in the Chinese context. Second, the existing literature ignores the role of ownership type in the relationship between executive compensation incentive and innovation capability, and we find that SOEs have a greater impact of long-term equity incentive on innovation capability than POEs. This paper could extend our understanding by examining the impact of executive compensation incentive on innovation capability in SOEs and POEs. Finally, the findings could help managers to design a reasonable incentive system in order to stimulate managers’ innovation, thus maintaining the sustainable development of agro-based companies.

The remainder of this paper is as follows. Section 2 presents the literature review and develops the testable hypotheses. Section 3 presents the methodology, and Section 4 reports and discusses the empirical results. Finally, Section 5 concludes the paper.

## 2. Literature review and hypotheses development

### 2.1 Principal-agent theory

Jensen and Meckling [2] proposed the classic principal-agent theory. They believed that the separation of ownership and management rights results in the principal-agent relationship between shareholders and executives, but this relationship also causes the principal-agent problem due to asymmetric information and incomplete company contracts. On the one hand, as a Homo economicus, both the principal and the agent aim to maximize their own interests. In terms of business operation, the agents usually pay more attention to their own efforts, while the principals focus more on the results of operations, which separates the interests of them. On the other hand, there is information asymmetry between the principal and the agent. This asymmetry can be divided into pre-event asymmetry and post-event asymmetry in chronological order. Before shareholders hire the agents, they do not know the manager’s ability, so they could only provide compensation according to the average market level. Competent managers would withdraw from the market when they meet this situation, while shareholders would further reduce the average salary when they hire the competent managers, which would cause the “lemon effect”. After shareholders hire managers, because of the unverifiability of managers’ efforts and the information asymmetry between production and operations, managers will use their own information advantages to maximize their interests, which tends to result in moral hazard and adverse selection behavior. The principal-agent theory emphasizes that company contracts are incomplete, and the incentive mechanism uses incentive contracts as a governance mechanism to supplement incomplete company contracts. This mechanism is not only a risk-sharing mechanism, but also a mechanism for property right allocation. Incentive contracts can effectively reduce the principal-agent problem. Thus, compensation contracts can affect the behavior of executives in the process of innovation.

Regarding corporate innovation, there exists a serious principal-agent problem [18]. Shareholders have deeply recognized that innovation is the core competitiveness of a company and the fundamental to its survival [19]. Therefore, shareholders tend to encourage technological innovation, while corporate executives may not support it [20]. The monetary compensation of executives in China is closely related to current firm performance [21]. However, technological innovation has its special characteristics with lagged returns [5]. It can be inferred that corporate innovation can lead to a short-term decline in firm performance, which directly affects the salary of executives in the current year.

### 2.2 Executive compensation incentive and innovation capability

The incentive level of executive compensation is closely related to corporate financial performance. The better the firm’s financial performance, the higher the executive compensation. However, executives generally focus on corporate short-term profits, ignore corporate long-term interests and technological innovation, and agree with the concept of “a bird in the hand”, resulting in the conflicts of interest between executives and shareholders [6]. In order to alleviate this contradiction, shareholders adopt compensation incentive measures to urge executives to better serve the company, attach importance to technological innovation, and promote the long-term development of companies. In the Chinese context, executives are still in the primitive stage of capital accumulation, and shareholders’ short-term compensation incentive is particularly important to them.

Senior management as corporate innovation subject participates in innovation activities by making major operational decisions and detailed work plans. However, because of high risks of innovation activities, senior executives are required to have appropriate professional sensitivity to make rapid response to market changes and timely adjust the business plan, which plays a key role in the process of corporate innovation. According to the principal-agent theory, executives may maintain their own interests at the expense of their shareholders through the sub-optimal decision-making. In order to alleviate this contradiction, shareholders will implement long-term equity incentives for executives and optimize the distribution of profits.

Based on the expectation theory, when executives decide whether or not to support innovation, they will consider the expected outcomes brought by innovation activities. However, from the perspectives of managers, supporting innovation means taking on the risks of high costs and failure rates [22]. Once the innovation activities fail, shareholders will think that managers do not work hard. However, in fact, they might put a lot of efforts into corporate innovation. Therefore, the higher the failure rate, the more likely executives are to resist innovation [22]. However, if executives hold shares in the company, they integrate the roles of managers and shareholders. When considering the expected results of supporting innovation, they as corporate shareholders can receive some benefits of innovation, such as the improvement of core competitiveness and the rise in stock prices. In this way, it can alleviate executives’ resistance to innovation. In addition, at the company level, innovation theory holds that entrepreneurs are the main body of innovation activities. With the separation of ownership and management rights, executives have taken on almost all the responsibilities of entrepreneurs. Therefore, establishing a reasonable incentive mechanism to motivate executives can effectively promote corporate innovation.

Based on the data from Chinese private enterprises, He and Jiang [1] found that executive compensation incentive is beneficial to increase innovation investment. A study by Zulfiqar and Hussain [23] showed a positive relationship between chief executive officer (CEO) compensation and firm innovation. The findings of Bintarto et al. [24] revealed that compensation given to executives motivates them to invest more in R&D activities. Zhou et al. [6] argued that executives’ salary has a positive impact on firms’ R&D investment, while equity incentive fails to promote R&D investment. Therefore, the following hypotheses are proposed as follows:

*Hypothesis 1 (H1)*. There is a positive relationship between short-term monetary incentive and corporate innovation capability.
*Hypothesis 2 (H2)*. There is a positive relationship between long-term equity incentive and corporate innovation capability.

Domestic and foreign scholars have long been discussing the relationship between executive compensation and corporate innovation. Xu [25] pointed out that the extant research mainly focuses on three perspectives, namely, direct relevance perspective, dynamic contingency perspective, and system integration perspective. The first perspective holds that there is a simple linear or non-linear relationship between executive compensation incentive and technological innovation. The dynamic contingency perspective is to analyze the interaction between them under different situations. The system integration perspective suggests that different forms of executive compensation incentive should be combined in the literature that investigates the interaction effects between different incentive methods. Using the data of listed companies in China’s growth enterprise market (GEM), Hu and Ma [26] concluded that there is an interactive relationship between salary incentive and equity incentive in promoting corporate innovation, and the optimal compensation incentive structure depends on the integration of different incentive methods. Therefore, the following hypothesis is proposed as follows:

*Hypothesis 3 (H3)*. There is a complementary effect between short-term monetary incentive and long-term equity incentive, and a reasonable compensation structure can effectively promote innovation capability.

### 2.3 Executive compensation incentive and innovation capability under different ownership types

There is a complex principal-agent relationship in SOEs. On the one hand, China is a socialist country with public ownership economic system, and has absolute control over SOEs [27]. Under this special institutional background, senior executives of SOEs are administratively appointed. They only care about the economic benefits of companies in order to pave the way for their future career. In addition, the administrative evaluation system has some defects, and the relevant institutional arrangements are not reasonable, which restricts the innovation capability of SOEs. On the other hand, because the scale of SOEs is relatively large, equity incentive is seldom used to motivate executives in order to guarantee the absolute control of SOEs.

However, in POEs, the performance of senior executives is closely linked to corporate profits. In order to obtain more income, senior executives must strive to improve firm performance. Superior firm performance can make them get equity incentive. In addition, executives in POEs are more likely to put emphasis on corporate innovation because scientific and technological innovation is the driving force of corporate sustainable development in today’s dynamic business environment [28]. Based on the above arguments, the following hypotheses are proposed as follows:

*Hypothesis 4 (H4)*. The impact of short-term monetary incentive on corporate innovation capability in POEs is greater than that in SOEs.
*Hypothesis 5 (H5)*. The impact of long-term equity incentive on corporate innovation capability in POEs is greater than that in SOEs.
*Hypothesis 6 (H6)*. Short-term monetary incentive and long-term equity incentive have mutually complementary effects on corporate innovation capability, and their complementary effects are greater in POEs than in SOEs.

## 3. Methodology

### 3.1 Sample selection and data collection

This paper takes agro-based companies listed on the Shanghai and Shenzhen stock exchanges from 2012 to 2019 as the research sample. The COVID-19 pandemic broke out in 2020, and it had a serious effect on corporate business [29]. It was reported by the Governance Institute of Australia that executive salaries were greatly reduced during the COVID-19 pandemic. Therefore, the time period of 2020–2022 is not included in our study. We exclude companies with abnormal and missing data, companies listed after 2012, delisted companies, and special treatment (ST) companies. The final sample of this paper includes 203 companies with 1624 observations. The data are sourced from the China Stock Market & Accounting Research (CSMAR) database and the RESSET database. The regressions are carried out using SPSS Version 22.

### 3.2 Variables

1. Dependent variable. Guided by He and Jiang [1], Jiang and Wang [17], Jin et al. [27], and Xu et al. [30, 31], we use R&D intensity (measured by the ratio of R&D investment to sales revenue) to measure corporate innovation capability.
2. Independent variables. Monetary incentive of executives is short-term salary income. Following Jiang and Wang [17] and Zhang et al. [32], this paper uses the ratio of the average compensation of top three executives to sales revenue as the measurement of short-term monetary incentive. The shareholding ratio of senior executives is equity or stock option incentive, which belongs to long-term non-monetary income. In this paper, the proportion of senior executives’ shareholding is used to measure long-term equity incentive, which is consistent with Zhang et al. [32] and He and Chen [33].
3. Control variables. Based on the existing literature [17, 27, 31, 33–35], this paper uses company size (SIZE), capital structure (CS), return on equity (ROE), and operating profit rate (OPR) as control variables. SIZE is measured through the natural logarithm of total assets at year-end. CS is measured by the ratio of total liabilities to total assets. ROE is measured by dividing net income by net assets. OPR is the ratio of operating profit to sales.

Table 1 shows the definition of all variables in this study.

**Table 1:** Variable definition.

Variable type	Variable	Symbol	Variable definitions
Dependent variable	Innovation capability	RD	R&D investment/sales revenue
Independent variables	Short-term monetary incentive	SMI	Average compensation of top three executives/sales revenue
	Long-term equity incentive	LEI	Number of shares held by senior management staff/total number of shares
Control variables	Company size	SIZE	Natural logarithm of total assets
	Capital structure	CS	Total liabilities/total assets×100%
	Return on equity	ROE	Net income/net assets×100%
	Operating profit rate	OPR	Operating profit/sales revenue×100%

### 3.3 Models

To test the impact of short-term monetary incentive on innovation capability, Model (1) is established as follows:

$$RD_{i,t}=\beta_{0}+\beta_{1}SMI_{i,t}+\beta_{2}SIZE_{i,t}+\beta_{3}CS_{i,t}+\beta_{4}ROE_{i,t}+\beta_{5}OPR_{i,t}+\varepsilon_{i,t}$$ (1)

To test the impact of long-term equity incentive on innovation capability, Model (2) is established as follows:

$$RD_{i,t}=\beta_{0}+\beta_{1}LEI_{i,t}+\beta_{2}SIZE_{i,t}+\beta_{3}CS_{i,t}+\beta_{4}ROE_{i,t}+\beta_{5}OPR_{i,t}+\varepsilon_{i,t}$$ (2)

In order to establish a reasonable incentive system, it is necessary to clarify the relationship between short-term monetary incentive and long-term equity incentive. The interaction SMI×LEI is introduced in Model (3).

$$RD_{i,t}=\beta_{0}+\beta_{1}SMI_{i,t}+\beta_{2}LEI_{i,t}+\beta_{3}SMI_{i,t}\times LEI_{i,t}+\beta_{4}SIZE_{i,t}+\beta_{5}CS_{i,t}+\beta_{6}ROE_{i,t}+\beta_{7}OPR_{i,t}+\varepsilon_{i,t}$$ (3)

where *i* is the firm; *t* represents the year; *β* stands for the presumed parameter; *ε* denotes the measurement error term.

## 4. Results and discussion

### 4.1 Descriptive statistics

Table 2 shows the descriptive statistics of all variables in full sample, SOEs, and POEs, respectively. The mean RD (0.01605) in full sample indicates that R&D investment level of agro-based companies in China is relatively lower than that of manufacturing companies, which is in line with the findings of Xu and Zhang [36]. Regarding company ownership, POEs have higher level of innovation capability than SOEs. It is generally believed that when R&D intensity reaches 5%, a company can obtain competitive advantage [27]. With regard to short-term monetary incentive (SMI), the mean value in SOEs and POEs is 0.00037 and 0.00056, respectively, indicating that POEs tend to have more salary incentives than SOEs to stimulate their executives. In terms of long-term equity incentive (LEI), the mean value in SOEs is 0.01114, while it is 0.17640 in POEs. The significant difference in LEI between the two sub-groups suggests that POEs may have a large minority shareholding. In addition, SOEs, on average, are larger and have more debt and higher performance.

**Table 2:** Descriptive statistics.

Variable	Full sample		SOEs		POEs	
	Mean	SD	Mean	SD	Mean	SD
RD	0.01605	0.02524	0.01152	0.01435	0.01888	0.02977
SMI	0.00049	0.00124	0.00037	0.00050	0.00056	0.00153
LEI	0.11288	0.20153	0.01114	0.05318	0.17640	0.23182
SIZE	9.45170	0.42388	9.53290	0.46831	9.40104	0.38543
CS	38.43665	20.05690	42.72291	19.07970	35.76203	20.20192
ROE	3.89403	8.00593	3.40159	7.56165	4.20130	8.26215
OPR	2.43097	63.99891	4.94999	30.60618	0.85910	77.88050

Based on the Shapiro-Wilk test, all variables do not have the normal data distribution (*p* < 0.05). This suggests that further analysis should use Pearson’s correlation coefficient analysis.

### 4.2 Correlation analysis

Table 3 presents the results of correlation analysis. There is a significant positive correlation between LEI and RD, indicating that the more equity the executives hold, the higher level of corporate innovation capability, which preliminarily proves H2. However, SMI is not significantly correlated with RD. The interaction effect between SMI and LEI is positively correlated with RD. In addition, we compute the variance inflation factors (VIFs) and find them all to be less than 5, implying that multi-collinearity is not a serious issue in this paper.

**Table 3:** Correlation matrix.

Variable	RD	SMI	LEI	SMI×LEI	SIZE	CS	ROE	OPR
RD	1							
SMI	0.031	1						
LEI	0.242***	0.000	1					
SMI×LEI	0.290***	0.147***	0.674***	1				
SIZE	-0.071**	-0.298******	-0.122***	-0.169***	1			
CS	-0.196***	-0.047	-0.157***	-0.177***	0.191***	1		
ROE	0.052*	-0.136***	0.059*	0.024	0.270***	-0.421***	1	
OPR	0.046	-0.289***	0.039	0.029	0.159***	-0.126***	0.436***	1

* *p* < 0.10

** *p* < 0.05

*** *p* < 0.01.

### 4.3 Regression results

Table 4 shows the regression results of Model (1). In full sample, the coefficient of SMI is positive, but it is not significant at the 5% level. Therefore, H1 cannot be verified. Firm performance is an important source of executive income. Higher payment to executives cannot effectively disperse risks, and the risk of shareholders can be dispersed through diversified mode of operation. Therefore, executives are cautious about R&D investment. Once R&D activities fail to bring high returns, companies would suffer great losses because of their high risks [5]. Tosi [37] and Hu et al. [38] also confirmed this conclusion. Additionally, He [39] found that CEO inside debt compensation promotes high financial reporting quality. However, Onishi [40] found that monetary compensation plans for employee inventions can result in an increase in the number of high cited patents in Japan. Lu et al. [3] concluded that there is an inverted U-shaped relationship between executive salary incentive and R&D investment. In addition, CS exerts a negative and significant on RD, while other control variables have no significant impact. In terms of ownership, the coefficient of SMI in SOEs is negative but insignificant (β = -0.491, t = -0.287). However, in POEs, SMI has a positive but insignificant impact on RD (β = 0.748, t = 0.884). Compared with SOEs, short-term monetary incentive in POEs has a better effect on corporate innovation capability.

**Table 4:** Regression results of Model (1).

Variable	Full sample	SOEs	POEs
Constant	0.037* (1.935)	0.029 (1.614)	0.025 (0.773)
SMI	0.489 (0.720)	-0.491 (-0.287)	0.748 (0.884)
SIZE	-0.001 (-0.560)	-0.001 (-0.654)	0.001 (0.213)
CS	-0.0003*** (-5.632)	-0.0001*** (-2.790)	-0.0003*** (-4.678)
ROE	-0.0001 (-1.184)	0.0001 (0.509)	-0.0003* (-1.716)
OPR	0.00002 (1.439)	-0.00002 (-0.729)	0.00003* (1.794)
Adj. R^2^	0.037	0.018	0.035
F	8.858***	2.416**	5.512***
N	1624	624	1000

* *p* < 0.10

** *p* < 0.05

*** *p* < 0.01. *t*-values are in parentheses.

Table 5 shows the regression results of Model (2). In full sample, the coefficient of LEI is positive and significant (β = 0.027, t = 7.024), indicating that long-term equity incentive can improve corporate innovation capability. Therefore, H2 is verified. According to the principal-agent theory, giving executives effective equity incentive can not only take into account the interests of the principals and reduce the principal-agent cost, but also protect their interests to the maximum extent and stimulate their desire for technological innovation, thus improving firm performance. Chen et al. [41] found that executive equity-based compensation can alleviate R&D overinvestment in the US software industry. However, Chen and Qian [42] observed that fixed compensation accounts for a relatively high proportion in the compensation design of Chinese listed companies, and long-term equity incentive do not function well.

**Table 5:** Regression results of Model (2).

Variable	Full sample	SOEs	POEs
Constant	0.024 (1.313)	0.024 (1.471)	0.019 (0.631)
LEI	0.027*** (7.024)	0.034** (2.549)	0.024*** (4.859)
SIZE	-0.0002 (-0.103)	-0.001 (-0.455)	0.001 (0.238)
CS	-0.0002*** (-4.997)	-0.0001*** (-2.844)	-0.0003*** (-4.252)
ROE	-0.0002 (-1.279)	0.0001 (0.400)	-0.0003 (-1.598)
OPR	0.00001 (1.121)	-0.00002 (-0.688)	0.00002 (1.337)
Adj. R^2^	0.082	0.034	0.070
F	19.096***	3.715***	10.297***
N	1621	623	998

** *p* < 0.05

*** *p* < 0.01. *t*-values are in parentheses.

In SOEs, the coefficient of LEI is positive and significant (β = 0.034, t = 2.549), suggesting that long-term equity incentive is significantly positively correlated with innovation capability. The higher the shareholding ratio of senior executives, the more the innovation investment. In POEs, there is also a positive relationship between LEI and RD (β = 0.024, t = 4.859). It is worth noticing that the impact of long-term equity incentive on innovation capability in SOEs is greater than that in POEs, which leads to the rejection of H5. Tsao et al. [43] suggested that the sensitivity of CEO compensation to R&D investment is higher in family firms than in non-family firms.

Table 6 presents the regression results of Model (3). In full sample, the coefficient of SMI×LEI is positive and significant at the 5% level, which supports H3. The findings of Zhang [44] showed that the overall level of executive compensation of Chinese agricultural listed companies is increasing year by year. Ikram et al. [45] pointed out that equity-based compensation exhibits varying return characteristics. It is necessary to design a reasonable compensation structure. Belloc [46] also believed that the combination of different incentive methods is optimal, and their synergistic effect on technological innovation can be achieved by examining the interaction between executive incentive methods. The coefficient of SMI×LEI in SOEs is positive and significant (β = 53.383, t = 2.205), and this coefficient in POEs is also positive and significant (β = 33.234, t = 4.123). Interestingly, the interaction effect in SOEs is greater than that in POEs, which rejects our H6.

**Table 6:** Regression results of Model (3).

Variable	Full sample	SOEs	POEs
Constant	0.015 (0.812)	0.034* (1.884)	-0.001** (-0.044)
SMI	-0.017 (-0.025)	-2.242 (-1.215)	0.281 (0.335)
LEI	0.010* (1.877)	0.012 (0.734)	0.007 (1.095)
SMI×LEI	33.901*** (5.113)	53.383** (2.205)	33.234*** (4.123)
SIZE	0.001 (0.312)	-0.002 (-0.919)	0.003 (0.831)
CS	-0.0002*** (-4.598)	-0.0001*** (-2.954)	-0.0003*** (-3.862)
ROE	-0.0001 (-1.098)	0.00004 (0.354)	-0.0003 (-1.467)
OPR	0.00001 (0.964)	-0.00001 (-0.256)	0.00002 (1.242)
Adj. R^2^	0.104	0.041	0.093
F	17.839***	3.390***	10.152***
N	1621	623	998

* *p* < 0.10

** *p* < 0.05

*** *p* < 0.01. *t*-values are in parentheses.

### 4.4 Robustness check

We use the ratio of R&D investment to total assets to measure innovation capability and the natural logarithm of total sales as a proxy for firm size, and re-estimate Models (1)-(3). In addition, we also use 1-year lagged SMI and LEI to re-estimate all models. The results are similar to previous findings, which suggests that our conclusion is robust.

## 5. Conclusions

This paper takes agro-based companies listed on the Shanghai and Shenzhen stock exchanges during 2012–2019 as the research sample, and investigates the impact of executive compensation incentive on corporate innovation capability. The empirical results show that short-term monetary incentive is not conducive to corporate innovation, while long-term equity incentive can promote innovation investment. The impact of long-term equity incentive on innovation capability in SOEs is greater than that in POEs. In addition, regardless of company ownership, compensation incentive structure is significantly correlated with corporate innovation capability.

The theoretical contributions of this paper are as follows. First, it expands the existing literature by exploring the impact of executive compensation incentive structure on innovation investment under different ownership types. Second, this paper might provide new insights for agribusiness managers to deeply understand the role of different compensation plans. In addition, as Chinese economy is the fastest growing developing economy, this study could be selected as a benchmark for other developing countries to guide companies to develop a reasonable and effective executive compensation incentive system.

The findings of this paper have several practical implications for corporate managers. Firstly, agro-based companies should establish a reasonable incentive system and change the single mode of salary incentive, which can avoid the problems of moral hazard and adverse selection of executives and effectively alleviate the contradiction between principals and agents. When incentivizing executives, such companies should consider the combination of financial and non-financial indicators instead of simply depending on the current financial performance. In addition, SOEs should implement long-term equity incentive and connect the interests of executives and shareholders. It is evidenced that the innovation that comes from private companies becomes a very high percentage of China’s innovation [47]. For POEs, they should increase incentives for executive and enhance their sense of belongings within the company in order to devote themselves to their work. Meanwhile, SOEs and POEs should not ignore the short-term effect of monetary incentive. Secondly, in China’s agricultural sector, human capital plays a vital role in corporate sustainable development [48], and agro-based companies should improve the incentive mode and give full play to the role of professional talents in innovative activities. According to the specific situation and corporate salary system, such companies should give certain salary incentives to talents to fully stimulate their vitality. Finally, at present, the executives of agro-based companies mainly focus on the short-term effects of technological innovation and ignore its long-term interests. These companies should encourage executive to be more engaged in R&D activities and support their innovative ideas in order to achieve sustainable development.

The limitations of this paper are as follows. First, this paper only focuses on agro-based companies. For generalizability, future research can extend the sample to other industries. Second, some factors such as market competition [49] influencing compensation incentive may not be included in regression model, and future research could include other internal and external factors. Additionally, considering that this study only uses R&D intensity to measure innovation capability, future research could involve patent information [50] as an alternative measure.
